# Co-Infections of Tilapia Lake Virus, *Aeromonas hydrophila* and *Streptococcus agalactiae* in Farmed Red Hybrid Tilapia

**DOI:** 10.3390/ani10112141

**Published:** 2020-11-18

**Authors:** Lukman Basri, Roslindawani Md. Nor, Annas Salleh, Ina Salwany Md. Yasin, Mohd Zamri Saad, Nor Yasmin Abd. Rahaman, Timothy Barkham, Mohammad Noor Azmai Amal

**Affiliations:** 1 Aquatic Animal Health and Therapeutics Laboratory, Institute of Bioscience, Universiti Putra Malaysia, UPM Serdang 43400, Selangor, Malaysia; lukmanbasri95@gmail.com (L.B.); annas@upm.edu.my (A.S.); salwany@upm.edu.my (I.S.M.Y.); mzamri@upm.edu.my (M.Z.S.); 2 Department of Veterinary Laboratory Diagnosis, Faculty of Veterinary Medicine, Universiti Putra Malaysia, UPM Serdang 43400, Selangor, Malaysia; roslindawani@yahoo.com (R.M.N.); noryasmin@upm.edu.my (N.Y.A.R.); 3 Department of Aquaculture, Faculty of Agriculture, Universiti Putra Malaysia, UPM Serdang 43400, Selangor, Malaysia; 4 Department of Laboratory Medicine, Tan Tock Seng Hospital, Singapore 308433, Singapore; timothy_barkham@ttsh.com.sg; 5 Department of Biology, Faculty of Science, Universiti Putra Malaysia, UPM Serdang 43400, Selangor, Malaysia

**Keywords:** co-infections, Tilapia Lake Virus, *Aeromonas hydrophila*, *Streptococcus agalactiae*, tilapia

## Abstract

**Simple Summary:**

Tilapia is a freshwater fish that is commercially cultured around the world. However, intensification of tilapia culture often results in diseases, occasionally with co-infections of multiple pathogens. This paper reports the first case of red hybrid tilapia that naturally co-infected with Tilapia Lake Virus (TiLV), *Aeromonas hydrophila* and *Streptococcus agalactiae* in Malaysia. In January 2020, a tilapia farm in Selangor, Malaysia, reported a mass mortality of adult red hybrid tilapias, with 70% mortality. Bacterial isolation, PCR and sequencing analysis confirmed the presence of TiLV, *A. hydrophila* and *S. agalactiae* in the affected fish. As tilapia is widely cultured throughout the world, detection of multiple infections might signal a potential threat to the industry.

**Abstract:**

A high death rate among red hybrid tilapias was observed in a farm in Selangor, Malaysia, in January 2020. The affected fish appeared lethargic, isolated from schooling group, showed loss of appetite, red and haemorrhagic skin, exophthalmia and enlarged gall bladders. Histopathological assessment revealed deformation of kidney tubules, and severe congestion with infiltrations of inflammatory cells in the brains and kidneys. Syncytial cells and intracytoplasmic inclusion bodies were occasionally observed in the liver and brain sections. Tilapia Lake Virus (TiLV), *Aeromonas hydrophila* and *Streptococcus agalactiae* were identified in the affected fish, either through isolation or through PCR and sequencing analysis. The phylogenetic tree analysis revealed that the TiLV strain in this study was closely related to the previously reported Malaysian strain that was isolated in 2019. On the other hand, *A. hydrophila* and *S. agalactiae* were closer to Algerian and Brazilian strains, respectively. The multiple antibiotic resistance index for *A. hydrophila* and *S. agalactiae* was 0.50 and 0.25, respectively. Co-infections of virus and bacteria in cultured tilapia is a new threat for the tilapia industry.

## 1. Introduction

Tilapia (*Oreochromis* sp.) is a commonly cultured freshwater fish around the world, with a total production of 6.51 million metric tonnes (MT) in 2016 [1]. The production of tilapia is dominated by China, followed by Indonesia, Egypt, Brazil, Philippines, Thailand and Bangladesh. In Malaysia, tilapia serves as the second-highest harvested freshwater fish, with an annual production of 25,199 MT, at a wholesale value of approximately USD 61.5 million. Tilapia production is expected to increase in the future as this species can withstand an intensive culture system [2].

Worldwide tilapia production is currently affected by a newly emerging disease caused by Tilapia Lake Virus (TiLV) [3]. In fact, TiLV was first reported in Israel [4], and subsequently in several Asian countries including Thailand, Indonesia and Malaysia [5,6,7]. TiLV is an enveloped single-stranded RNA virus, which consists of 10 unique genomic segments. Only the first segment shared a weak similarity with Orthomyxoviridae virus [8]. However, TiLV has now been officially recognised as a novel virus under the genus *Tilapinevirus* and family Amnoonviridae, and scientifically known as *Tilapia tilapinevirus* [9]. TiLV is contagious and it has been associated with ‘summer mortality syndrome’ in Egypt and ‘tilapia one-month syndrome’ in Thailand, where it causes 20–90% mortality [5,10].

Aeromoniasis and streptococcosis are common bacterial diseases affecting tilapia culture, specifically involving *Aeromonas hydrophila* and *Streptococcus agalactiae* [11,12,13,14]. Apart from tilapia (*Oreochromis* sp.) culture, *A. hydrophila* and *S. agalactiae* were also found infecting other cultured fish species such as catfish (*Clarias* sp.) and golden pompano (*Trachinotus blochii*), respectively [15,16]. Infections caused by both bacterial pathogens also lead to massive fish mortalities and economic losses around the world [13,14].

Co-infections involving virus and bacteria are common in nature, including fish culture [17]. However, little attention has been given towards this phenomenon. Several cases of co-infections involving viral and bacterial pathogens have been reported in cultured tilapia, including *Iridovirus*-*Flavobacterium columnare*–*A. veronii*-*S. agalactiae* [18] and Infectious Spleen and Kidney Necrosis Virus (ISKNV)–*S. agalactiae* infections [19]. To date, numerous studies have also discovered co-infections involving TiLV with other bacterial species including *A. veronii* [7,20], *A. hydrophila*, *A. jandeii* [20], *Flavobacterium* sp. and *Streptococcus* sp. [5]. Occasionally, co-infections between *A. hydrophila* and *S. agalactiae* in tilapia were also reported [21,22]. Previous studies suggested that co-infections involving viral and bacterial pathogens might aggravate the disease and increase mortality in the affected fish [7,17,23]. In this study, we report the first case of co-infections of cultured red hybrid tilapia with TiLV, *A. hydrophila* and *S. agalactiae*.

## 2. Materials and Methods

### 2.1. Case History

In January 2020, a tilapia farm in Selangor, Malaysia, reported a mass mortality of adult red hybrid tilapias (*Oreochromis niloticus* × *O. mossambicus*). The affected fish were between 300 to 500 g body weight, and 20 to 30 cm body length. Between 50 to 100 fish died daily, over three consecutive weeks. The tilapia fry and water source in this farm was obtained from a local fish hatchery and nearby small river, respectively. All fish were cultured in earthen ponds and only tilapia was cultured here. The fish were cultured until they reached between 800 to 1000 g of body weight, before being supplied to the local fish markets and restaurants. The mean ± standard deviation (SD) of water quality during this study such as temperature, dissolved oxygen, pH and ammonia-nitrogen was recorded at 24.60 ± 0.80 °C, 4.27 ± 0.50 mg/L, 7.40 ± 0.20 and 0.34 ± 0.10 mg/L, respectively.

### 2.2. Samples Collection

A total of 20 morbid fish that showed either lethargic, isolated from schooling group, swim near the water surface, haemorrhagic skin and exophthalmia were randomly collected. The fish were immediately euthanized by pithing, and post-mortem examination was conducted. Tissues of the brain, kidney, liver and spleen were collected, pooled and preserved in RNAlater™ reagent (Invitrogen, Carlsbad, CA, USA) for virus RNA extraction and detection. For the bacterial isolation and identification, swabs of the eye, kidney, liver and spleen were streaked directly onto Tryptic Soy Agar (Merck, Darmstadt, Germany) with 5% horse blood, followed by incubation at 30 °C for 24 h. The brain, kidney, liver and spleen of the affected fish were also preserved in 10% buffered formalin (Sigma-Aldrich, St. Louis, MO, USA) for histopathological analysis.

### 2.3. Virus RNA Extraction and Detection

Total RNA of the pooled organs was extracted using TRIzol™ reagent (Invitrogen). A recombinant plasmid containing 415 bp of TiLV segment 3 was used as a positive control [24], while tissue samples of healthy tilapia from a non-affected farm were used as a negative control. The cDNA synthesis was carried out by using the Quantinova™ Reverse Transcription kit (Qiagen, Kuala Lumpur, Malaysia). TiLV detection was performed using semi-nested RT-PCR [24]. The first round of semi-nested RT-PCR’s mastermix consisted of 2 µL cDNA template, 0.4 µM of each primer Nested ext-1 (5′-TAT GCA GTA CTT TCC CTG CC-3′) and ME1 (5′-GTT GGG CAC AAG GCA TCC TA-3′), 5 µL of 5× buffer solution (Promega, Madison, WI, USA), 2.5 mM of MgCl2 (Promega), 0.4 mM of dNTP mix (Promega), 1U of Taq polymerase (Promega) and DNase-free water up to total volume of 25 µL. The second round of semi-nested RT-PCR was conducted in 20 µL reaction solution containing 1 µL of first-round semi-nested RT-PCR product, 0.25 µM of each primer 7450/150R/ME2 (5′-TAT CAC GTG CGT ACT CGT TCA GT-3′) and ME1 (5′-GTT GGG CAC AAG GCA TCC TA-3′), 5 µL of 5× buffer solution (Promega), 2.5 mM of MgCl2 (Promega), 0.4 mM of dNTP mix (Promega), 1U of Taq polymerase (Promega) and DNase-free water. PCR reaction was performed as follows: initial denaturation at 94 °C for 2 min, 25 cycles of denaturation at 94 °C for 30 s, annealing at 60 °C for 30 s and extension at 72 °C for 30 s, and a final extension at 72 °C for 5 min. The PCR product was subjected to gel electrophoresis in 2% agarose gel.

### 2.4. Bacterial Isolation, DNA Extraction and Identification

The bacteria to be identified were sub-cultured to get pure colonies before being subjected to Gram-stain, oxidase and catalase tests. Further identification was done using API^®^20E and API^®^20Strep kits (BioMérieux, Marcy-l’Étoile, France) according to the manufacturer’s recommendations.

Bacterial DNA was extracted using the Wizard^®^ Genomic DNA Purification Kit (Promega). Detection of *A. hydrophila* was performed using primers targeting the 16S rRNA gene of *A. hydrophila*, which were A.h 16S rRNA-F (5′-AGG TTG ATG CCT AAT ACG TA-3′) and A.h 16S rRNA-R (5′-CTG GCT GGC AAC AAA GGA CAG-3′) [25]. The PCR mixture contained 2 µL DNA template, 0.4 µM of each primer, 5 µL of 5× buffer solution (Promega), 2.5 mM of MgCl_2_ (Promega), 0.4 mM of dNTP mix (Promega), 1U of Taq polymerase (Promega) and DNase-free water up to the total volume of 25 µL. Amplification was carried out as follows: initial denaturation at 94 °C for 5 min, 35 cycles of denaturation at 95 °C for 30 s, annealing at 58 °C for 30 s and extension at 72 °C for 30 s, and a final extension at 72 °C for 10 min. DNA template from healthy fish was used as a negative control. Amplified products were visualized under UV light following electrophoresis in 2% agarose gel.

Amplification for *S. agalactiae* was performed using specific primers targeting the *cpsG* gene, which consisted of *cpsG*-F (5′-ACA TGA ACA GCA GTT CAA CCG T-3′) and *cpsG*-2-3-6-R (5′-TCC ATC TAC ATC TTC AAT CCA AGC-3′) [26]. The PCR mixture consisted of 2 µL DNA template, 0.4 µM of each primer, 5 µL of 5× buffer solution (Promega), 2.5 mM of MgCl_2_ (Promega), 0.4 mM of dNTP mix (Promega), 1U of Taq polymerase (Promega) and DNase-free water in a final volume of 25 µL. The amplification conditions consisted of initial denaturation at 94 °C for 5 min, 25 cycles of denaturation at 95 °C for 1 min, annealing at 56 °C for 1 min and extension at 72 °C for 2 min, and a final extension at 72 °C for 10 min. DNA template from healthy fish was used as a negative control. Amplified PCR products were visualized in 2% agarose gel.

### 2.5. Antibiotic Sensitivity Test

The antibiotic sensitivity of representative *A. hydrophila* (n = 3) and *S. agalactiae* (n = 3) that were isolated from this outbreak was evaluated using the Kirby–Bauer disk-diffusion susceptibility method [27]. Eight antibiotic disks (Oxoid, London, UK) were used, including ampicillin (10 µg), cefotaxime (30 µg), cefepime (30 µg), chloramphenicol (30 µg), ciprofloxacin (5 µg), gentamicin (10 µg), tetracycline (30 µg) and sulfamethoxazole/trimethoprim (1.25/23.75 µg). The resistance profiles were interpreted based on the recommended criteria of CLSI [28]. The multiple antibiotic resistance (MAR) index was determined according to Krumperman [29]. A MAR index of greater than 0.2 indicated high exposure towards these antibiotics.

### 2.6. Virus and Bacteria Sequencing

Purified PCR products from TiLV (n = 3), *A. hydrophila* (n = 3) and *S. agalactiae* (n = 3) were sequenced (First Base Laboratories, Kuala Lumpur, Malaysia). The nucleotide sequences were then compared with other known sequences in the GenBank using the Nucleotide Basic Local Alignment Search Tool (BLAST) program. Phylogenetic trees of TiLV, *A. hydrophila* and *S. agalactiae* were generated by using Neighbour-joining of the Mega 7 software [30].

### 2.7. Histopathological Assessment

Organs that were fixed in 10% buffered formalin were dehydrated, embedded in paraffin, sectioned at 4 µM (Leica Jung Multi cut 2045, Germany) and stained with haematoxylin and eosin (H&E). The slides were then examined under light microscope (Nikon, Minato City, Japan) to assess the histopathological changes.

## 3. Results

### 3.1. Disease Characterisation

**Figure 1:**
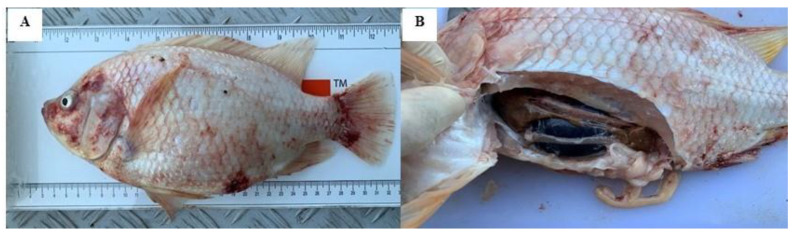
Clinical signs and gross lesions of red hybrid tilapia naturally co-infected by Tilapia Lake Virus, *Aeromonas hydrophila* and *Streptococcus agalactiae*. (**A**) Red skin with haemorrhages at the operculum and base of the caudal fin. (**B**) Enlarged gall bladder and darkening of liver.

This outbreak resulted in 70% mortality, affecting the red hybrid tilapias of the same batch and source, while other batches were not affected. The affected fish showed lethargy with slow movement, isolated from the schooling group, and appeared to swim near the water surface. There were haemorrhages on the skin (Figure 1A) and exophthalmia. On post-mortem examination, enlarged gall bladder was frequently observed (Figure 1B).

### 3.2. Bacteria and Virus Identification and Sequencing Analysis

**Figure 2:**
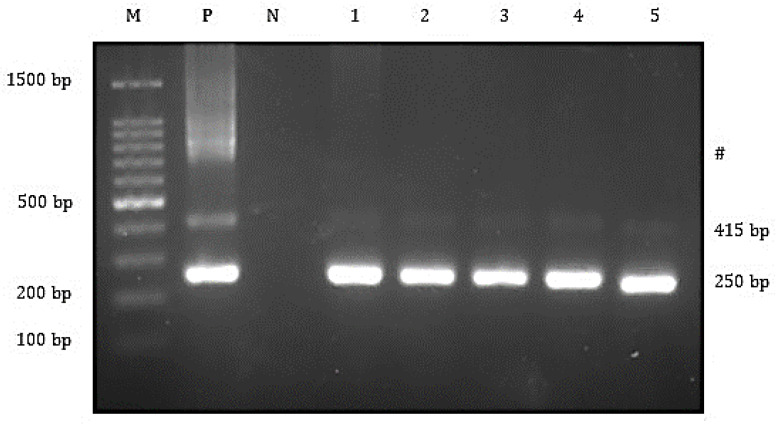
Agarose gel electrophoresis showing the 415 and 250 bp bands of the TiLV genes from the affected fish. Lane M = 1 kb DNA marker; Lane P = positive control; Lane N = negative control; Lane 1 to 5 = All TiLV-positive samples yielded 415 and 250 bp amplicons. #Marks band are from cross-hybridizations between amplified products.

**Figure 3:**
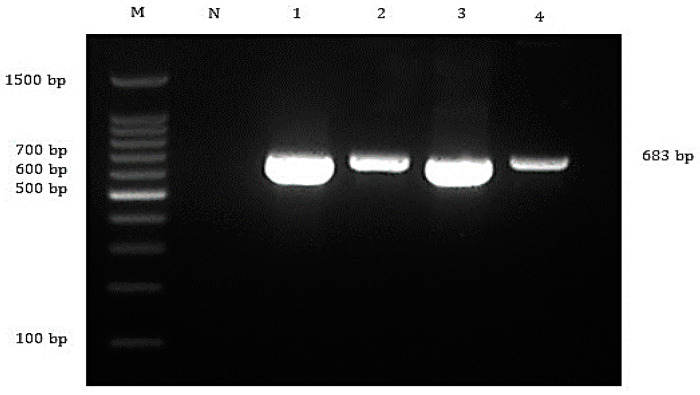
Agarose gel electrophoresis showing detection of 683 bp of *Aeromonas hydrophila* targeting 16S rRNA gene from the affected fish. Lane M = 1 kb DNA marker; Lane N = negative control; Lane 1 to 4 = All *A. hydrophila*-positive samples yielded 683 bp amplicons.

**Figure 4:**
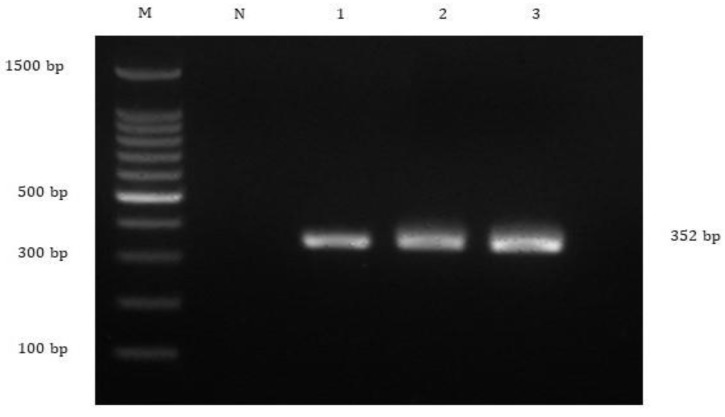
Agarose gel electrophoresis showing detection of 352 bp of *Streptococcus agalactiae* targeting *cpsG* gene from the affected fish. Lane M = 1 kb DNA marker; Lane N = negative control; Lane 1 to 3 = All *S. agalactiae*-positive samples yielded 352 bp amplicons.

Biochemical tests revealed the presence of *A. hydrophila* and *S. agalactiae* in the affected fish (Supplementary Materials Tables S1 and S2). The subsequent PCR revealed that 100% (20/20) of fish were positive for TiLV and *A. hydrophila* (Figure 2 and Figure 3), while *S. agalactiae* was detected in 50% (10/20) of the affected fish (Figure 4). Therefore, 50% of the sampled fish were simultaneously infected with TiLV, *A. hydrophila* and *S. agalactiae*, while the remaining 50% were simultaneously infected with TiLV and *A. hydrophila*.

**Figure 5:**
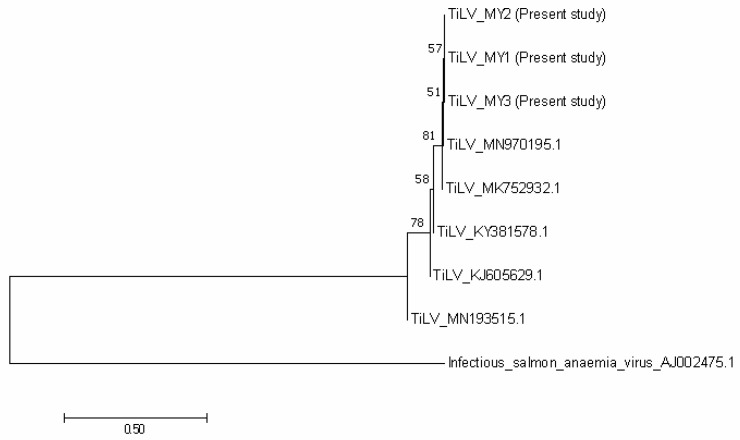
Phylogenetic tree showing the relationship between the three TiLV sequences from the present study, and other reference sequences from Malaysia (MN970195.1), India (MK752932.1), Thailand (KY381578.1), Israel (KJ605629.1) and the United States (MN193515.1). The tree is based on the 250 bp of TiLV’s segment 3. The infectious salmon anaemia virus was considered as an out group.

**Figure 6:**
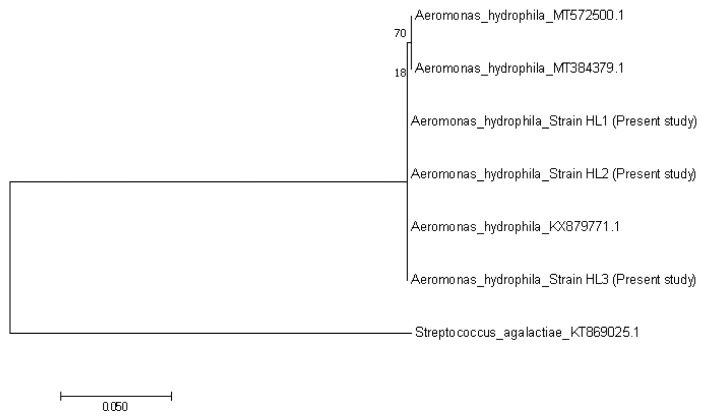
Phylogenetic tree showing the relationship between the three *Aeromonas hydrophila* sequences from the present study, with other reference sequences from Algeria (MT572500.1), India (MT384379.1) and Iran (KX879771.1). The tree is based on the 683 bp 16S rRNA gene of *A. hydrophila*. *Streptococcus agalactiae* was considered as an out group.

**Figure 7:**
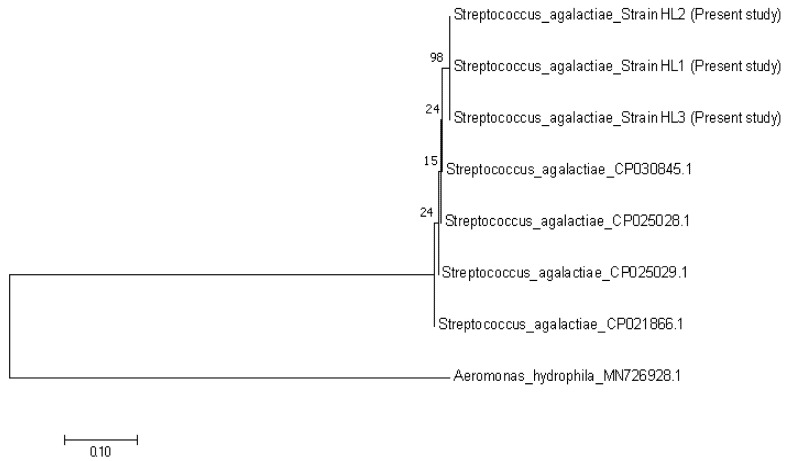
Phylogenetic tree showing the relationship between the three *Streptococcus agalactiae* sequences from the present study, with other reference sequences from Brazil (CP030845.1) and Singapore (CP025028.1, CP025029.1 and CP021866.1). The tree is based on the 352 bp *cpsG* gene of *S. agalactiae*. *Aeromonas hydrophila* was considered as an out group.

The nucleotide sequences of TiLV revealed 99% homology to the previous Malaysian strain that was isolated in 2019 (MN970195.1). The three representatives of TiLV strains that were obtained in this study were grouped together and closer with the strains from Malaysia (MN970195.1) and India (MK752932.1), than the strains from Thailand (KY381578.1), Israel (KJ605629.1) and the United States (MN193515.1) (Figure 5). Furthermore, nucleotide sequences of *A. hydrophila* in this study showed 97% similarity with the published sequence of *A. hydrophila* strain IR-Kh-Ah-93 from Iran (KX879771.1). The phylogenetic tree showed that the three representatives of *A. hydrophila* from the present study were grouped together with *A. hydrophila* from Iran (KX879771.1), compared with other *A. hydrophila* strains from Algeria (MT572500.1) and India (MT384379.1) (Figure 6). Nucleotide sequences of *S. agalactiae* showed 99% similarity with the *S. agalactiae* strain S73 (CP030845.1) from Brazil. The representative *S. agalactiae* strains of this study were closely related with the Brazilian strain (CP030845.1), compared with the Singaporean strains (CP025028.1, CP025029.1, and CP021866.1) (Figure 7).

### 3.3. Antibiotic Sensitivity Test

*Aeromonas hydrophila* was found to be resistant to sulfamethoxazole/trimethoprim, ampicillin, gentamicin and ciprofloxacin, but susceptible to tetracycline, cefepime, cefotaxime and chloramphenicol (Table 1). On the other hand, *S. agalactiae* was found to be resistant to gentamicin and tetracycline, but intermediately susceptible to ciprofloxacin. They were highly susceptible with sulfamethoxazole/trimethoprim, cefepime, ampicillin, cefotaxime and chloramphenicol. The MAR index of *A. hydrophila* and *S. agalactiae* was 0.50 and 0.25, respectively.

### 3.4. Histopathological Assessment

**Figure 8:**
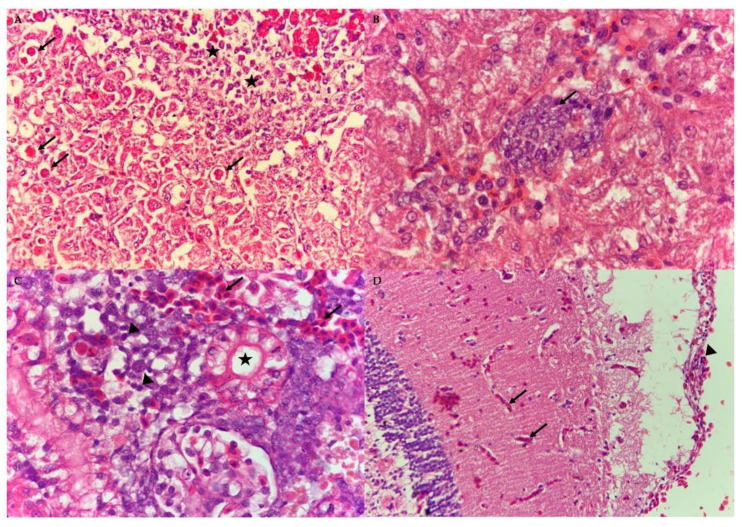
Photomicrograph of the liver, kidney and brain of the naturally co-infected red hybrid tilapia by Tilapia Lake Virus, *Aeromonas hydrophila* and *Streptococcus agalactiae*. (**A**) Infiltration of lymphocytic inflammatory cells (stars) and eosinophilic intracytoplasmic inclusion bodies (arrows) in the liver section (40×, H&E). (**B**) Syncytial giant cell exhibiting multiple nuclei (arrow) in the liver section (100×, H&E). (**C**) Mild renal tubular degeneration (star) and haemorrhages (arrows) in the kidney section together with moderate infiltration of mononuclear inflammatory cells (arrow heads) (100×, H&E). (**D**) Mild congestion (black arrows) and infiltration of lymphocytes (arrowhead) in the meninges (40×, H&E).

Histopathological assessment revealed severe congestion and necrosis in the livers, together with infiltration of numerous inflammatory cells (Figure 8). Diffused eosinophilic intracytoplasmic inclusion bodies in the hepatocytes and occasional syncytial giant cells were observed in the liver sections of the infected fish. The kidney showed mild to moderate haemorrhages and tubular degeneration and necrosis, with infiltration of mononuclear inflammatory cells in the interstitial space. The brain, cerebral and meningeal haemorrhages and congestion were noted, along with lymphocytic meningitis. Many melano-macrophage centres were observed in the spleen.

## 4. Discussion

Intensification of tilapia culture often results in exposing tilapia towards multiple diseases, often with co-infections [31]. This study discovered a natural co-infection of TiLV, *A. hydrophila* and *S. agalactiae* in cultured red hybrid tilapia. Earlier, there was a report of cultured tilapias infected with TiLV that were also positive to *Flavobacterium* sp. and *Aeromonas* sp. [5]. Similarly, previous studies also found co-infections of TiLV and *A. veronii* in red hybrid tilapia juveniles [7], while *A. sobria* and *Staphylococcus xylosus* had concurrently been isolated in wild river carps (*Barbonymus schwanenfeldii*) that were positive to TiLV [32].

Isolation, PCR and sequencing analysis confirmed the presence of TiLV, *A. hydrophila* and *S. agalactiae* in the current outbreak. Moreover, the TiLV strain in this study was closely related with the previous TiLV isolated in Malaysia in 2019. The phylogenetic tree also revealed that the Malaysian isolates were closely related to the Indian strain, which might indicate the possibility of the disease spreading between continents [24]. According to Dong et al. [33], TiLV could have existed unnoticed a long time ago in cultured tilapia, and worldwide trading of fish fry spread the organism. The recent intensification of tilapia rearing leads to the emergence of TiLV infections. On the other hand, *A. hydrophila* in this study was closer to the Iranian strain and shared similar phenotypic and biochemical characteristics with *A. hydrophila* isolated from Nile tilapia (*O. niloticus*) from Egypt [34]. Furthermore, isolates of *S. agalactiae* in this study were found closer to the Brazilian isolate and have similar characteristics with *S. agalactiae* isolated from tilapia in Thailand, with the exception of the arginine dihydrolase test [35]. Like TiLV, *A. hydrophila* and *S. agalactiae* isolates in this study were found to be closely related with strains from other countries, indicating that the organisms had spread across the continents during importations of the fish fry [33]. However, further study should be conducted to confirm this.

The gross lesions and histopathological assessment of the affected fish in this study resembled common findings of TiLV-, *Aeromonas-* and *Streptococcus*-infected fish [7,14,36,37]. Syncytial giant cells and multinucleated cells were observed in the liver and brain sections, that were previously described in the TiLV-infected and co-infected fish [7,38]. Syncytial giant cell is a common histopathological feature found in the TiLV-infected fish, mostly observed in the liver [38]. Besides, intracytoplasmic inclusion bodies were also previously described in both natural and experimental TiLV-infected fish [38,39]. Haemorrhages, infiltration of inflammatory cells, deformation of kidney tubules and formation of melano-macrophages centres were observed in this outbreak and were commonly found in other co-infected fish involving TiLV, *Aeromonas* sp. and *Streptococcus* sp. [5,7,20]. Moreover, in this study, the gross lesions of co-infected fish were found to be more severe than fish with a single infection [36].

Infections by TiLV, *A. hydrophila* and *S. agalactiae* might have a synergistic effect that resulted in increased severity of the disease, leading to a high rate of mortality. Similarly, higher mortality rate (25–100%) was also observed during TiLV outbreaks involving secondary bacterial infections in Malaysia, Thailand and Egypt [5,7,20]. Fish infected by TiLV alone, usually, but not necessarily, showed lower rates of mortalities of 9.2% in Egypt [10], 6.4% in Chinese Taipei [40], 15% in Malaysia [41] and 2.71% in Mexico [42]. A study by Nicholson et al. [43] supported the high rate of mortality following co-infections when they reported a mortality rate of 93% among TiLV–*A. hydrophila* co-challenged fish, and 34% mortality among single-challenged fish with TiLV and 6.7% with *A. hydrophila*. Nevertheless, three (15%) of fish samples collected from this farm also exhibited no clinical signs or gross lesions, indicating the possibility of inapparent infection, and the infected fish possibly developed specific immunity against the pathogens.

The MAR index of *A. hydrophila* and *S. agalactiae* indicated high exposure of these pathogens to antimicrobial agents, and the possible sources should be further investigated. However, frequent use of antimicrobial agents eventually give rise to antibiotic resistance among the fish, leading to difficulty in controlling diseases in the future [44].

## 5. Conclusions

In this study, we reported the first case of co-infections of red hybrid tilapia with TiLV, *A. hydrophila* and *S. agalactiae* in Malaysia. Multiple infections in tilapia might signal a potential threat to the Malaysian aquaculture industry. Thus, future research should be conducted to better understand the synergistic effects of co-infections with TiLV, *A. hydrophila* and *S. agalactiae*, that could help in developing a polyvalent vaccine to combat these three important pathogens.

## Figures and Tables

**Table 1:** Antibiotic susceptibility of *Aeromonas hydrophila* and *Streptococcus agalactiae* isolated in red hybrid tilapia using the disk diffusion method.

Antibiotic	Disk Potency (µg)	*Aeromonas hydrophila* (Zone of Inhibition, mm)	*Streptococcus agalactiae* (Zone of Inhibition, mm)	Zone of Inhibition (mm)		
				R	I	S
Ampicillin	10	R (6)	S (20)	≤13	14–16	≥17
Cefepime	30	S (23)	S (25)	≤14	15–17	≥18
Cefotaxime	30	S (25)	S (23)	≤14	15–22	≥23
Ciprofloxacin	5	R (13)	I (19)	≤15	16–20	≥21
Chloramphenicol	30	S (29)	S (18)	≤12	13–17	≥18
Gentamicin	10	R (11)	R (10)	≤12	13–14	≥15
Sulfamethoxazole/trimethoprim	1.25/23.75	R (0)	S (20)	≤10	11–15	≥16
Tetracycline	30	S (15)	R (9)	≤11	12–14	≥15

Note: R = resistant, I = intermediate susceptible, S = susceptible.

## Supplementary Materials

The following are available online at https://www.mdpi.com/2076-2615/10/11/2141/s1, Table S1: Comparison of phenotypic and biochemical characteristics between isolated *Aeromonas hydrophila* in the present and previous study, Table S2: Comparison of phenotypic and biochemical characteristics between *Streptococcus agalactiae* in the present and previous studies.
