# Supplementary material for: Co-Infections of Tilapia Lake Virus, Aeromonas hydrophila and Streptococcus agalactiae in Farmed Red Hybrid Tilapia
Source: Animals (Basel). 2020 Nov 18;10(11):2141. doi: 10.3390/ani10112141 (PMC7698767; doi:10.3390/ani10112141)
Supplement: Supplementary file 1 [file animals-10-02141-s001.pdf]

Supplementary Table S1. Comparison of phenotypic and biochemical characteristics between isolated *Aeromonas hydrophila* in the present and previous study.

Characteristics	*A. hydrophila* from red hybrid tilapia (present study)	*A. hydrophila* from Nile tilapia [34]
Gram stain	- (rod)	- (rod)
Haemolysis	+	+
Oxidase	+	+
Catalase	+	+
Motility	+	+
β-galactosidase	+	NA
Arginine dihydrolase	+	+
Lysine decarboxylase	-	-
Ornithine decarboxylase	-	-
Citrate utilization	+	+
H_2S production	-	-
Urease	-	-
Tryptophane deaminase	-	-
Indole production	+	+
Acetoin production	+	NA
Gelatinase	+	+
Fermentation/oxidation of glucose	+	+
Fermentation/oxidation of mannitol	+	+
Fermentation/oxidation of inositol	+	+
Fermentation/oxidation of sorbitol	-	-
Fermentation/oxidation of rhamnose	-	-
Fermentation/oxidation of saccharose	+	NA
Fermentation/oxidation of melibiose	-	-
Fermentation/oxidation of amygdalin	-	-
Fermentation/oxidation of arabinose	+	+

*Note: + = positive, - = negative, NA = not available

34. Abdel-Latif, H.M.; Khafaga, A.F. Natural co-infection of cultured Nile tilapia *Oreochromis niloticus* with *Aeromonas hydrophila* and *Gyrodactylus cichlidarum* experiencing high mortality during summer. *Aquac. Res.* **2020**, *51*, 1880-1892.

Supplementary Table S2. Comparison of phenotypic and biochemical characteristics between isolated *Streptococcus agalactiae* in the present and previous study.

**Characteristics**	***S. agalactiae* from red hybrid tilapia (present study)**	***S. agalactiae* from tilapia [35]**
Gram stain	+ (cocci)	+ (cocci)
Haemolysis	+	+
Oxidase	-	-
Catalase	-	-
Motility	-	-
Acetoine production	+	+
Hydrolysis of hippuric acid	+	+
B-glucosidase hydrolysis	-	-
Pyrrolidonyl arylamidase	-	-
α-galactosidase	-	-
β-glucuronidase	-	-
β-galactosidase	-	-
Alkaline phosphate	+	+
Leucine aminopeptidase	+	+
Arginine dihydrolase	-	+
Acidification of ribose	+	+
Acidification of arabinose	-	-
Acidification of mannitol	-	-
Acidification of sorbitol	-	-
Acidification of lactose	-	-
Acidification of trehalose	+	+
Acidification of inulin	-	-
Acidification of raffinose	-	-
Acidification of amidon	-	-
Acidification of glycogen	-	-

*Note: + = positive, - = negative

35. Soto, E.; Wang, R.; Wiles, J.; Baumgartner, W.; Green, C.; Plumb, J.; Hawke, J. Characterization of isolates of *Streptococcus agalactiae* from diseased farmed and wild marine fish from the US Gulf Coast, Latin America, and Thailand. *J Aquat. Anim. Health* **2015**, *27*, 123-134.
